# Antibiotic treatment of respiratory tract infections in adults in Norwegian general practice

**DOI:** 10.1093/jacamr/dlac135

**Published:** 2023-01-07

**Authors:** Marius Skow, Guro H Fossum, Sigurd Høye, Jørund Straand, Louise Emilsson, Anja Maria Brænd

**Affiliations:** Department of General Practice, The Antibiotic Centre for Primary Care, Institute of Health and Society, University of Oslo, Oslo, Norway; Department of General Practice, Institute of Health and Society, University of Oslo, Oslo, Norway; Department of General Practice, The Antibiotic Centre for Primary Care, Institute of Health and Society, University of Oslo, Oslo, Norway; Department of General Practice, General Practice Research Unit (AFE), Institute of Health and Society, University of Oslo, Oslo, Norway; Department of General Practice, The Antibiotic Centre for Primary Care, Institute of Health and Society, University of Oslo, Oslo, Norway; Department of General Practice, General Practice Research Unit (AFE), Institute of Health and Society, University of Oslo, Oslo, Norway; Department of General Practice, Institute of Health and Society, University of Oslo, Oslo, Norway; Vårdcentralen Värmlands Nysäter and Centre for Clinical Research, County Council of Värmland, Varmlands Nysater, 661 95 Karlstad, Sweden; Department of Medical Epidemiology and Biostatistics, Karolinska Institutet, Stockholm, Sweden; School of Medical Science, University of Örebro, Örebro, Sweden; Department of General Practice, Institute of Health and Society, University of Oslo, Oslo, Norway

## Abstract

**Objectives:**

To analyse the prevalence of respiratory tract infection (RTI) episodes with and without antibiotic prescriptions in adult patients in Norwegian general practice during the period 2012–2019.

**Methods:**

Observational study linking data from the Norwegian Control and Payment for Health Reimbursements Database and the Norwegian Prescription Database. Episodes of acute RTIs in patients aged 18 years or older were identified and linked to antibiotic prescriptions dispensed within 7 days after diagnosis. We analysed annual infection rates and antibiotic prescription rates and antibiotics prescribed for the different RTI conditions.

**Results:**

RTI episode rate per 1000 inhabitants was 312 in 2012 and 277 in 2019, but showed no linear trend of change during the study period (*P* = 0.205). Antibiotic prescription rate decreased from 37% of RTI episodes in 2012 to 23% in 2019 (*P* < 0.001). The reduction in prescribing was most pronounced for episodes coded with ICPC-2 symptom diagnoses, as well as upper RTIs, influenza, acute bronchitis and sinusitis. Prescriptions for phenoxymethylpenicillin decreased from 178 746 in 2012 to 143 095 in 2019, but increased as proportion of total antibiotic prescriptions from 40% in 2012 to 53% in 2019 (*P* < 0.001).

**Conclusions:**

This study demonstrates stable RTI episode rates and reduced antibiotic prescription rates for RTIs for adults in Norwegian general practice 2012–2019. We also observed a shift towards relatively more use of phenoxymethylpenicillin and less broad-spectrum antibiotics. These changes are in line with the aims of the Norwegian strategy against antibiotic resistance.

## Introduction

Antimicrobial resistance (AMR) is a global threat to public health.^1^ Excessive consumption of antibiotics is a main driver for antibiotic resistance development at both individual and community levels.^2–4^ Antibiotic stewardship aims to reduce inappropriate and unnecessary use of antibiotics to reduce AMR and preserve the therapeutic effect of antibiotics. Norwegian health authorities launched a National Strategy for Antibiotic Resistance in 2015, aiming to reduce the national antibiotic consumption by 30% expressed as defined daily dose (DDD) per 1000 inhabitants per day (DID) and reduce antibiotic prescriptions for respiratory tract infections (RTIs) by 20% by year 2020 compared to 2012 levels.^5^ Following the national strategy, the health authorities facilitated several voluntary quality improvement projects aiming for more appropriate prescribing in Norwegian hospitals and general practice. Among these projects, an audit-based course for primary care doctors was implemented in 2018,^6^ based on methods proven to be effective in a primary care setting.^7,8^

In Norway, 84% of all antibiotics for human use are prescribed outside hospitals and nursing homes.^9^ Most of these prescriptions are usually attributed to general practitioners (GPs).^9^ Thus, GPs have a key role for the appropriateness of antibiotic use. RTIs account for approximately half of all antibiotic prescriptions in Norway.^9^ However, many of the RTIs seen in general practice are viral or self-limiting bacterial infections, and antibiotic treatment for mild infections, such as sinusitis and sore throat, have no or only a marginal effect.^10,11^ As well as prescribing an antibiotic when this is not warranted, a suboptimal choice of antibiotic is also considered as inappropriate antibiotic prescribing. The current national guidelines for Norwegian primary care recommend phenoxymethylpenicillin (PcV) as a first-line antibiotic for almost all acute RTIs,^12^ and beta-lactamase sensitive penicillin as percentage of the total antibiotic consumption is a national and European quality indicator.^13,14^ Studies on overprescribing in general practice have identified high proportions of inappropriate prescribing.^15–17^

From 2012 to 2019, Norway had achieved a 22% decrease in total human antibiotic consumption based on sales statistics from primary care, hospitals and nursing homes.^9^ In 2019, the human antibiotic consumption in the community was 13.6 DID, while the European mean was 18.0 DID.^18^ Norway has a low ratio of broad- to narrow-spectrum antibiotics at 0.1, compared with the European mean ratio at 2.8.^18^

To inform clinicians, policy makers and stewardship programmes, and to better understand the changes in antibiotic use, it is essential to monitor both the number of patients seeking their GP for infections, and the corresponding antibiotic prescriptions. In this study we aimed to analyse the prevalence of RTI episodes in adult patients treated in Norwegian general practice and the rate of dispensed antibiotic prescriptions, DDD per prescription and type of antibiotic for RTI treatment during the period 2012–2019.

## Materials and methods

### Ethics

The study was approved by the Regional Committee for Medical and Health Research Ethics, REC South East (ref. 2016/2283), and the Norwegian Data Protection Authority (ref. 282558). The study was conducted in accordance with the Declaration of Helsinki and institutional standards.

### Design and setting

We conducted an observational study linking data from nationwide health registries. The study period was from 2012 to 2019. All Norwegian residents have access to public healthcare through the National Insurance Scheme and are assigned to a regular GP. In 2015, 99% of the population was assigned to a regular GP list, and most of the Norwegian population use their GP or the municipal GP-staffed out-of-hours (OOH) services when seeking medical help.^19^ In Norway, antibiotics are available through prescription only.

### Data sources

The Norwegian Control and Payment for Health Reimbursements (KUHR) Database receives compensation claims from GPs and OOH services.^20^ For each claim, the database contains ID for patient and physician, date of contact and one or two diagnoses per contact according to the International Classification of Primary Care (ICPC-2).^21^ We obtained claims for all registered contacts (consultations by attendance, telephone contacts and e-consultations) from GPs and OOH services with an infection related ICPC-2 diagnosis. The Norwegian Prescription database (NorPD) includes detailed information on all prescription drugs dispensed at Norwegian pharmacies.^22^ We obtained the following data from NorPD: encrypted IDs for patients and prescribers, prescription date, prescribed drug item categorized according to the Anatomical Therapeutic Chemical Classification (ATC) system, and DDD of prescription in accordance with the ATC/DDD index of 2019.^23^ Statistics Norway routinely collects demographic and geographic data on the Norwegian population.^24^ We extracted data on patient sex, and month and year of birth and death (if applicable). Data from the registries were linked using the unique personal identification number (encrypted by the NorPD algorithm) assigned to all Norwegian residents. All databases cover the entire Norwegian population.

### Population

We included all patients aged ≥18 years with a relevant diagnosis (see Table S1, available as Supplementary data at *JAC-AMR* online) recorded during 2012–2019. We selected adult patients as both treatment guidelines and clinical decision-making in paediatric patients differ from adults. Patients were included from 1 January 2012. Further patients were included each month after their 18th birthday, along with new registrants in the KUHR database, and follow-up ended at study completion (31 December 2019) or the month of the patient’s death.

### RTI episode definition and antibiotic prescriptions

As each patient could have more than one GP contact during the course of an infection, we investigated episodes of acute RTIs rather than consultations. The episode start date (index date) was defined as the date of diagnosis if no RTI diagnoses had been recorded within the previous 30 days. If there were less than 30 days between diagnoses, the latter diagnosis was defined as a re-consultation and assigned to the initial episode. The 30-day period was based on previous comparable studies on RTI episodes.^25–28^ For each acute RTI episode, follow-up ended 90 days after the index date. If an episode had more than one of the included diagnoses recorded during the follow-up, we selected the diagnosis most likely to receive an antibiotic prescription as main diagnosis (Table S1). Episodes were defined as symptom-related or infection-specific according to the ICPC-2 classification system, where codes R01-29 are used for respiratory symptoms/complaints, and R71-81 and R83 are used for particular RTIs. Episodes with infectious diagnoses not related to the respiratory tract but which may indicate antibiotic treatment (e.g. urinary tract infections, skin- and soft tissue infections), were excluded.

We included antibacterial agents for oral use (ATC code J01) excluding methenamine, nitrofurantoin, (piv)mecillinam and trimethoprim, because in Norway, these antibiotics are exclusively prescribed for urinary tract infections. For each RTI episode, we analysed the first antibiotic prescription dispensed within 7 days following a GP consultation. Antibiotic classes were grouped as tetracyclines (ATC code J01AA), PcV (J01CE02), other penicillins (J01C except J01CE02), macrolides (J01FA) and other antibiotics.

### Statistical analyses

Descriptive statistics were calculated with means and proportions for each year and for the entire study period. Age was analysed in groups from 18–24 years, then 10-year age groups up to >85 years. Annual RTI episode rates were calculated by dividing the number of episodes by number of inhabitants ≥18 years in the year of index date. To account for changes in the population, rates were age-and-sex-standardized using direct standardization using the Norwegian 2012 population as reference. The study mean RTI episode rates presented are the mean of yearly episode rates for the 8 year study period. Prescription rate was calculated as the proportion of episodes resulting in at least one dispensed antibiotic prescription. Relative proportions of the different antibiotic classes were calculated.

For annual trends, we used linear regression with standardized yearly RTI episode rates over year for all diagnoses individually, and for total RTI episodes. Linear regression over year was also conducted for yearly prescription rate, DDD per prescription and proportion of each antibiotic class. Coefficients from the regressions are presented as mean yearly change with 95% CI.

To test for differences in mean contacts per episode, we used the Mann–Whitney *U*-test to compare sexes and non-parametric ANOVA (Kruskal–Wallis test) to compare age groups. Chi-squared tests were used to compare differences between sexes and between age groups in prescription rates and penicillin proportion. As we observed different patterns in episode rates for age groups between men and women, we ran a linear regression with yearly episode rate over age group for each sex to test for linear trends.

The significance level was set to 0.05. Means are presented with standard deviation (SD). STATA/SE v.16.1 (StataCorp LLC) was used for all calculations.

## Results

### RTI episodes

During the study period, 2 931 421 adult patients were diagnosed with at least one RTI in general practice. At first consultation, 54% were women, mean age was 45.5 years (SD 19.5) (Table 1). Sex and age distribution did not change substantially over time. In total, 14 209 411 RTI contacts were registered during the study period. According to our definition, this amounted to 9 181 118 acute RTI episodes. Seven out of ten episodes, 70% (6 397 673) had only one consultation, 18% (1 692 388) had two, while 12% (1 091 057) had three or more consultations. Mean number of contacts per RTI episode was 1.5 (SD 1.1) for men and 1.6 (SD 1.2) for women (*P* < 0.001), and did not change noticeably during the study period.

**Table 1.** Acute RTI episodes in adult patients treated in Norwegian general practice 2012–2019

	Total	Females	Males
Patients	2 931 421	1 591 663	1 339 758
%		54%	46%
Mean age (SD)	45.5 (19.5)	45.6 (19.8)	45.3 (19.2)
RTI consultations	14 209 411	8 541 093	5 668 318
RTI episodes	9 181 118	5 422 569	3 758 549
Mean number of consultations per episode (SD)	1.5 (1.1)	1.6 (1.2)	1.5 (1.1)
Mean annual RTI episodes per 1000 inhabitants	284	335	232
By age group			
18–24	326	395	258
25–34	286	353	218
35–44	278	337	218
45–54	263	317	208
55–64	283	340	226
65–74	276	307	245
75–84	300	306	293
85+	311	277	344
By infection (six most common diagnoses)			
URTI (R74)^a^	65	80	52
Sinusitis (R75)^a^	26	37	16
Tonsillitis (R72 + R76)^a^	12	15	10
Acute bronchitis (R78)^a^	27	32	22
Pneumonia (R81)^a^	21	22	21
Influenza (R80)^a^	19	21	17

a ICPC-2 codes.

**Figure 1.**
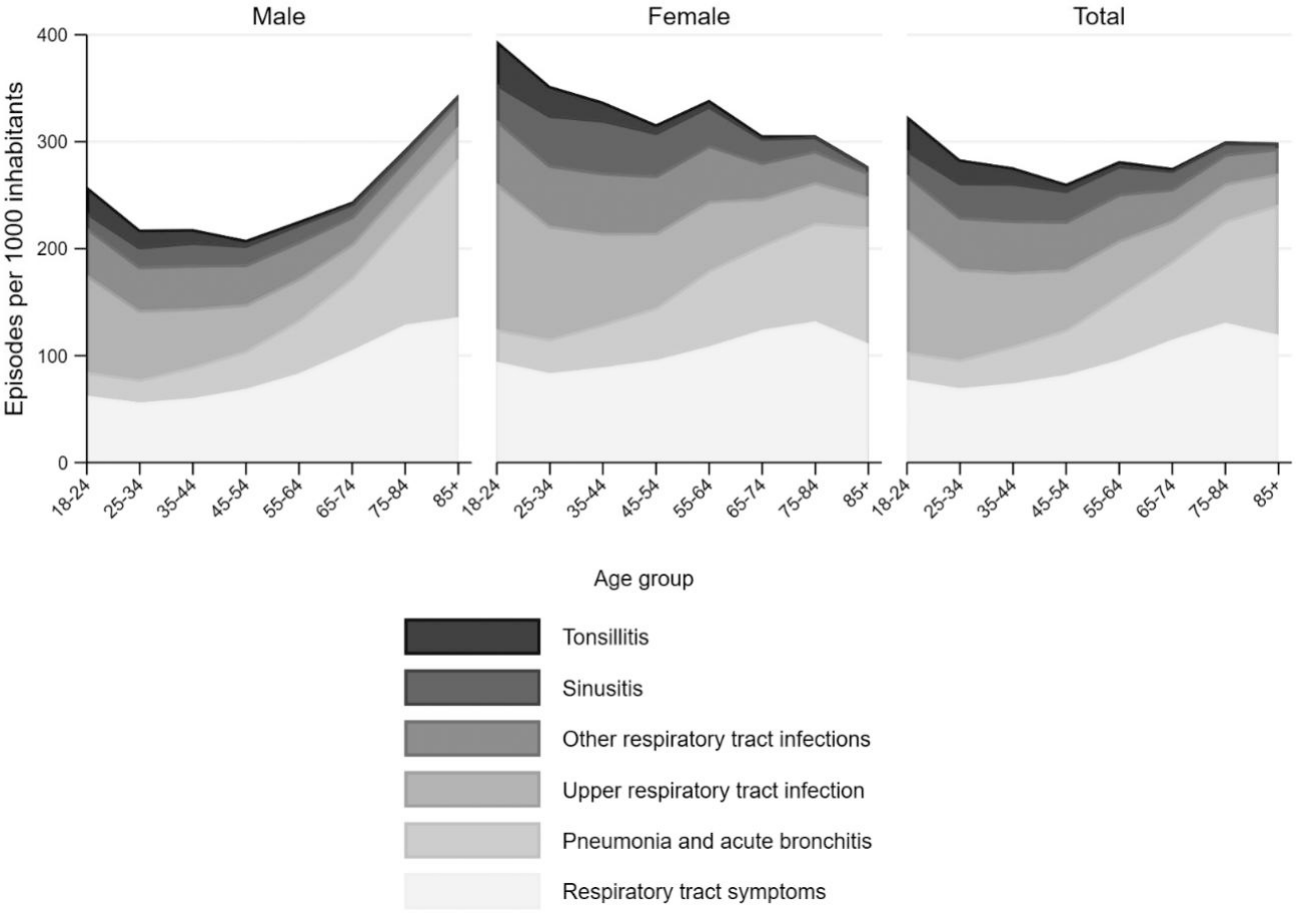
Age and sex distribution of mean annual acute respiratory tract episodes per 1000 adult inhabitants per year in Norwegian general practice 2012–2019.

Women had more episodes than men (Table 1), and the number of yearly RTI episodes per 1000 inhabitants varied between age groups for both men and women, with episode rates increasing by age for men (*P* of linear trend <0.001) and decreasing by age for women (*P* of linear trend <0.001) (Figure 1). The number of consultations per episode increased with age, from 1.4 (SD 0.9) consultations per episode in the youngest age group, to 1.7 (SD 1.4) in the oldest age group (*P* for difference <0.001). Mean age of patients with pneumonia was 61.7 years (SD 19.3), for sinusitis 43.8 years (SD 15.4), for upper respiratory tract infection (URTI) 41.1 years (SD 17.4), and for tonsillitis 33.4 years (SD 13.1).

There were 1 205 782 RTI episodes in 2012, and 1 165 545 in 2019. Accounting for population changes, the age- and sex-standardized yearly RTI episode rates per 1000 adult inhabitants were 312 in 2012 and 277 in 2019. However, we did not observe a significant linear decrease (*P* = 0.205) (Table 2). URTI (ICPC code R74) was the most commonly used diagnosis, and the use of this diagnosis increased during the study period (*P* of linear trend = 0.003). The use of all other infection-specific ICPC-2 codes except acute bronchitis (R78), influenza (R80) and other RTI (R83) decreased (Table S1). Symptom-related diagnoses (R1–29) accounted for 30% of all episodes in 2012 and 33% of all episodes in 2019.

**Table 2.** Respiratory tract infection episode rates and corresponding antibiotic prescription rates for the six most common RTI diagnoses and the corresponding symptoms in adult patients in Norwegian general practice 2012–2019

	Study mean	2012	2013	2014	2015	2016	2017	2018	2019	Mean annual change^a^ (95% CI)
**All RTIs**										
Episode rate^b^	284	312	285	266	284	284	281	280	277	−2.6 (−7.1 to 1.9)
Antibiotic prescription rate^c^	29	37	34	33	30	28	25	23	23	−2.1 (−2.4 to −1.7)^d^
PcV proportion (%)^e^	47	40	44	45	46	48	51	52	53	1.8 (1.5 to 2.1)^d^
**Diagnosis-specific ICPC-2 codes**										
Episode rate	193	218	197	179	193	193	191	192	187	−2.6 (−6.4 to 1.3)
Antibiotic prescription rate	37	45	42	42	38	36	33	30	31	−2.3 (−2.8 to −1.9)^d^
PcV proportion (%)	48	42	46	46	47	50	53	54	55	1.8 (1.5 to 2.2)^d^
**Symptom-related ICPC-2 codes**										
Episode rate	90	93	87	87	91	91	90	88	91	−0.1 (−0.9 to 0.8)
Antibiotic prescription rate	12	17	14	13	12	11	9	9	8	−1.2 (−1.4 to −1.0)^d^
PcV proportion (%)	35	30	33	35	35	36	37	38	39	1.2 (0.9 to 1.4)^d^
**URTI (R74)**										
Episode rate	65	63	59	58	63	65	70	71	75	2.2 (1.1 to 3.3)^d^
Antibiotic prescription rate	18	24	22	21	19	17	15	14	14	−1.6 (−1.9 to −1.3)^d^
PcV proportion (%)	56	50	54	54	55	57	60	62	62	1.7 (1.3 to 2.1)^d^
**Sinusitis (R75)**										
Episode rate	26	32	30	27	27	25	24	22	22	−1.5 (−1.7 to −1.2)^d^
Antibiotic prescription rate	57	65	64	61	59	55	51	49	49	−2.7 (−3.1 to −2.3)^d^
PcV proportion (%)	50	46	47	48	49	51	55	56	57	1.7 (1.3 to 2.2)^d^
**Sinus symptoms (R09)**										
Episode rate	5	5	5	5	5	6	6	5	6	0.2 (0.1 to 0.3)^d^
Antibiotic prescription rate	24	32	31	28	27	24	20	19	19	−2.1 (−2.5 to −1.8)^d^
PcV proportion (%)	44	41	42	42	43	43	46	47	48	1.0 (0.7 to 1.4)^d^
**Tonsillitis (R72 + R76)**										
Episode rate	12	15	13	13	12	12	12	11	11	−0.5 (−0.7 to −0.3)^d^
Antibiotic prescription rate	76	76	76	76	77	76	75	74	76	−0.2 (−0.4 to 0.0)
PcV proportion (%)	83	80	81	81	82	84	86	86	87	1.1 (0.9 to 1.3)^d^
**Throat symptoms (R21)**										
Episode rate	15	16	15	15	15	15	15	14	15	−0.1 (−0.2 to 0.1)
Antibiotic prescription rate	15	20	19	18	17	15	13	12	11	−1.3 (−1.5 to −1.2)^d^
PcV proportion (%)	66	64	64	66	66	67	69	69	70	0.8 (0.7 to 1.0)^d^
**Acute bronchitis (R78)**										
Episode rate	27	31	26	26	30	30	28	24	22	−0.8 (−1.8 to 0.2)
Antibiotic prescription rate	44	57	53	50	45	41	36	34	36	−3.4 (−4.1 to −2.6)^d^
PcV proportion (%)	25	20	23	24	25	27	30	30	29	1.5 (1.0 to 2.0)^d^
**Pneumonia (R81)**										
Episode rate	21	29	23	21	22	21	21	19	17	−1.2 (−1.9 to −0.5)^d^
Antibiotic prescription rate	66	68	66	67	67	67	66	64	65	−0.4 (−0.6 to −0.1)^d^
PcV proportion (%)	40	34	38	38	40	42	44	45	45	1.6 (1.1 to 2.0)^d^
**Cough (R05)**										
Episode rate	37	41	37	35	37	37	37	35	34	−0.6 (−1.2 to 0.0)
Antibiotic prescription rate	13	20	16	15	14	12	10	9	9	−1.5 (−1.8 to −1.2)^d^
PcV proportion (%)	20	16	19	19	20	21	23	24	24	1.1 (0.9 to 1.3)^d^
**Influenza (R80)**										
Episode rate	19	19	22	13	20	20	18	26	16	0.2 (−1.3 to 1.7)
Antibiotic prescription rate	7	9	8	8	7	7	6	5	5	−0.6 (−0.7 to −0.5)^d^
PcV proportion (%)	41	35	39	39	39	43	43	45	44	1.2 (0.7 to 1.7)^d^

a Mean annual change represents coefficient from linear regression with 95% CI.

b Episode rate: episodes per 1000 inhabitants adjusted for age and sex.

c Antibiotic prescription rate: proportion of episodes receiving ≥1 prescription within 7 days.

d *P* value <0.05.

e PcV proportion (%): phenoxymethylpenicillin as proportion of all first prescriptions.

### Antibiotic prescriptions

Antibiotics were prescribed for 29% (2 659 088) of all RTI episodes. Most prescriptions (72%; 1 922 967) were dispensed after the first GP contact, and 17% (461 404) after the second contact of the episode. Prescriptions dispensed the same day as the GP consultation comprised 83% (2 201 251) of all, whereas 9% (245 619) were dispensed on day two and 8% (212 218) between day 3 and 7. The overall prescription rates varied between age groups, with the least prescribing (27.3% of episodes) in 45–54-year-olds and the most (30.7% of episodes) in 65–64-year-olds during the study period (*P* for difference of 0.001).

The antibiotic prescription rate declined from 37% (443 633/1 205 782) in 2012 to 23% (271 149/1 165 545) in 2019, corresponding to a 37% decrease throughout the study period (*P* of linear trend <0.001) (Table 2). Reduced yearly prescription rates were observed in all age groups for both men and women.

PcV accounted for 47% of the antibiotic prescriptions during the study period; followed by macrolides (22%) and tetracyclines (17%) (Table 3). Although the absolute number of episodes receiving PcV prescriptions decreased from 178 746 in 2012 to 143 095 in 2019, PcV as a proportion of all antibiotic prescriptions increased from 40% in 2012 to 53% in 2019 (*P* of linear trend <0.001) (Table 3). Macrolide use declined throughout the study period from 28% of all in 2012 to 15% in 2019 (*P* of linear trend <0.001).

**Table 3.** Dispensed prescriptions for acute RTIs adult patients in Norwegian general practice 2012–2019

	Total	2012	2013	2014	2015	2016	2017	2018	2019	Mean annual change^a^ (95% CI)
RTI episodes with ≥1 dispensed AB prescription	2 659 088	443 633	376 136	345 585	344 393	320 045	292 444	265 703	271 149	
Mean DDD per prescription (SD)	12.1 (5.3)	11.6 (5.1)	11.7 (5.2)	11.9 (5.3)	12.1 (5.3)	12.2 (5.3)	12.4 (5.5)	12.5 (5.5)	12.5 (5.4)	0.1 (0.1 to 0.1)^b^
**Phenoxymethylpenicillin J01CE02**										
Proportion of first prescriptions (%)	47	40	44	45	46	48	51	52	53	1.8 (1.5 to 2.1)^b^
Mean DDD per prescription (SD)	14.1 (4.5)	13.7 (4.3)	13.7 (4.3)	13.9 (4.5)	14.3 (4.6)	14.2 (4.6)	14.2 (4.6)	14.3 (4.7)	14.3 (4.6)	0.1 (0.1 to 0.1)^b^
**Other penicillins J01C (−CE02)**										
Proportion of first prescriptions (%)	11	9	10	10	11	11	11	12	12	0.5 (0.4 to 0.6)^b^
Mean DDD per prescription (SD)	9.1 (2.7)	9.0 (3.1)	9.1 (2.7)	9.2 (2.5)	9.1 (2.5)	9.1 (2.6)	9.2 (2.6)	9.2 (2.5)	9.4 (2.7)	0.0 (0.0 to 0.0)^b^
**Macrolides J01FA**										
Proportion of first prescriptions (%)	22	28	24	24	22	20	18	16	15	−1.8 (−2.0 to −1.5)^b^
Mean DDD per prescription (SD)	8.2 (2.5)	8.4 (3.3)	8.2 (3.5)	8.2 (3.5)	8.2 (3.3)	8.1 (3.1)	8.2 (4.2)	8.2 (3.6)	8.4 (3.8)	−0.0 (−0.0 to −0.0)^b^
**Tetracyclines J01A**										
Proportion of first prescriptions (%)	17	19	18	18	18	17	16	16	16	−0.5 (−0.7 to −0.3)^b^
Mean DDD per prescription (SD)	14.1 (6.4)	14.1 (6.1)	14.0 (6.3)	14.2 (6.5)	14.1 (6.3)	14.2 (6.4)	14.2 (6.6)	14.3 (6.8)	14.1 (6.9)	0.0 (0.0 to 0.0)^b^
**Other antibiotics**										
Proportion of first prescriptions (%)	4	3	4	4	4	4	3	4	4	0.0 (−0.1 to 0.1)
Mean DDD per prescription (SD)	7.5 (4.0)	7.5 (4.0)	7.5 (3.8)	7.6 (4.1)	7.6 (4.0)	7.6 (3.8)	7.6 (4.0)	7.6 (4.5)	7.6 (4.3)	0.0 (0.0 to 0.0)^b^

a Mean annual change represents coefficient from linear regression with 95% CI.

b *P* value <0.05.

The mean DDD per prescription for all antibiotics was 12.1 (SD 5.3) during the study period. Overall, mean DDD per prescription increased from 11.6 DDD (SD 5.1) in 2012 to 12.5 DDD (SD 5.4) in 2019 (*P* of linear trend <0.001). Mean DDD per prescription increased for PcV from 13.7 (SD 4.3) in 2012 to 14.3 (SD 4.6) in 2019 (*P* < 0.001) and for other penicillins from 9.0 (SD 3.1) in 2012 to 9.4 (SD 2.7) in 2019 (*P* < 0.001) (Table 3).

Younger patients were more frequently treated with PcV. Patients aged 18–24 years received PcV in 64% of first prescriptions, whereas corresponding figures for patients aged 75–84 years and older than 85 years were 35% and 39%, respectively (*P* for difference <0.001). Men received a slightly higher proportion of PcV compared with women, with 47% versus 46% of all prescriptions (*P* for difference <0.001).

The highest prescription rates were seen for tonsillitis (76%), pneumonia (66%) and otitis media (65%). From 2012 to 2019, the prescription rates decreased significantly for all infection-specific diagnoses except for tonsillitis and whooping cough (Table S1). The largest relative reduction was seen for URTI, influenza, acute bronchitis and sinusitis (Table 2).

**Figure 2.**
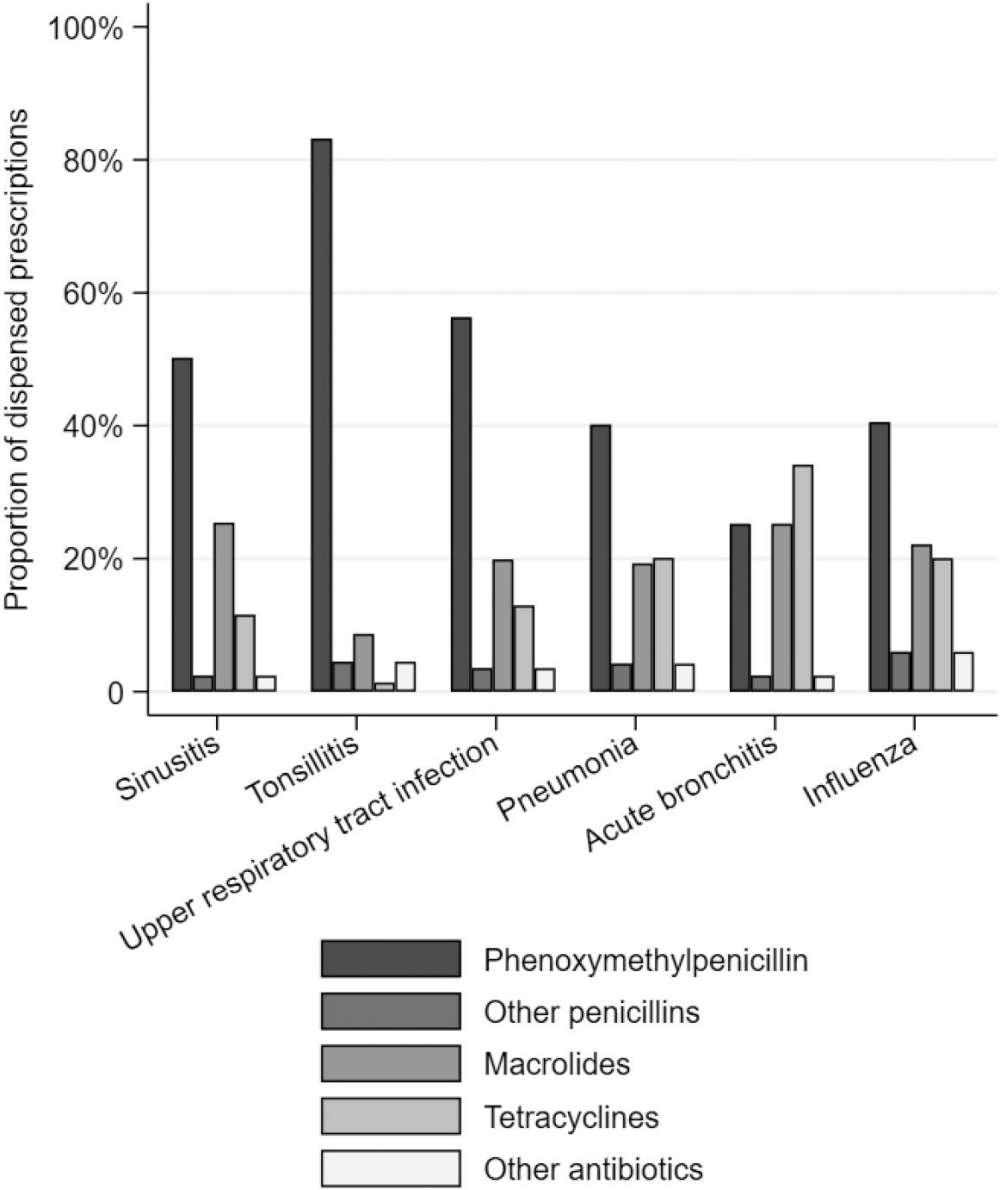
Antibiotic groups of dispensed prescriptions for the six most common RTI diagnoses in Norwegian general practice during 2012–2019.

Figure 2 illustrates differences in use of antibiotic classes between the six most common infection-specific diagnoses. Acute tonsillitis was almost exclusively treated with PcV (83% of prescriptions), macrolides were issued in one out of four patients with sinusitis or acute bronchitis, while tetracyclines were prescribed in 34% and 20% of prescriptions for acute bronchitis and pneumonia, respectively. PcV as a proportion of all prescriptions increased significantly for all infection-specific diagnoses except whooping cough and laryngitis/tracheitis (Table S1).

## Discussion

### Main findings

This registry-based study demonstrated a stable rate of acute RTI episodes and a decreased antibiotic prescription rate for RTIs in adults in Norwegian general practice between 2012 and 2019. The antibiotic prescription rate decreased by almost 40% in 2019 compared with 2012. Reduced prescribing rates were seen for all RTI diagnoses except tonsillitis and whooping cough. PcV was the most frequently prescribed antibiotic for RTIs, and a relative increase in PcV use was seen compared to other antibiotics.

### Comparison with other studies

Although we did not find a linear decrease in RTI episodes, we observed a change from 312 episodes per 1000 inhabitants in 2012 to 277 per 1000 in 2019. Norway experienced an epidemic of mycoplasma pneumoniae during the winter of 2011–2012,^29^ which explains a high episode rate and a relatively high proportion of macrolides issued in 2012. A recent Norwegian study covering 2006–2015 reported an increased number of consultations in primary care, but a decreased proportion of RTI consultations.^30^ Studies from Swedish general practice from the same decade reported decreased consultation rates for RTIs and a corresponding decrease in antibiotic prescription rates.^26,31^ Also, both Danish and English primary care research have reported decreased antibiotic prescription rates.^32,33^

Women had more RTI episodes and more contacts per episode than men, which is in line with findings from Swedish general practice.^34^ Interestingly, we observed that RTI episode rates increased with age for men, while decreasing with age for women. For both men and women, we observed relatively high RTI episode rates in the youngest age group mostly made up by patients aged 18 and 19 years, especially from 2016. This may reflect an absentee regulation for high school students implemented in 2016, urging students to see their GP to get a certificate of absence when unable to attend class.^35^ This may potentially contribute to antibiotic overuse, due to the positive association between RTI consultation rates and antibiotic prescription rates.^36,37^

Older patients had on average more consultations per episode, and were diagnosed with more severe infections than younger patients. However, we did not find higher antibiotic prescription rates in the oldest age groups, consistent with previous studies on antibiotic use in the community.^38^ An explanation for this is that our dataset did not include data from either hospitals or nursing homes. Elderly patients with more severe RTIs are more commonly treated in hospitals and about 40 000, mostly frail, are living in nursing homes.

We observed a relative increase in the use of symptom-related diagnoses (ICPC-2 R01-29) compared with infection-specific diagnoses (ICPC-2 R71-83) as well as increased use of the diagnosis URTI (R74). We assume that physicians tend to use more symptom-related diagnostic codes when deciding not to prescribe antibiotics and vice versa, on the basis of studies showing that the intention to prescribe influences the diagnostic labelling.^39^ However, although the reduction in antibiotic prescription rate was larger for symptom-related episodes, we saw a significant decrease in prescriptions for the infection-specific diagnoses.

The increased use of the URTI diagnosis could partly explain the reduction in sinusitis episodes, as URTI and sinusitis have overlapping symptoms and accurate diagnosing of bacterial sinusitis in general practice is challenging.^40^ We observed a decrease in sinusitis episode rates of 32%, combined with a reduction in antibiotic prescription rate of 26% while the proportion of PcV increased by 24%. Similar changes were observed for acute bronchitis. Both sinusitis and acute bronchitis have been specifically targeted by the Norwegian stewardship interventions in general practice.

Our finding that PcV was the most frequently prescribed antibiotic for RTIs is in line with studies from general practice in Sweden and Denmark.^26,41^ This is also reflected in the high proportions of narrow-spectrum penicillins out of the total antibiotic consumption in Scandinavia, with 21% in Norway, 27% in Denmark and 28% in Sweden.^14^ Although macrolide use decreased, it was still the second most commonly used antibiotic for RTIs accounting for 22% of prescriptions in the study period. This corresponds well with figures from Danish and British general practice.^41,42^ According to the Norwegian guidelines for antibiotic treatment of RTIs in primary care, macrolides are only recommended as first choice empirical treatment for atypical pneumonia, whooping cough and as an alternative to PcV in patients with penicillin allergy.^12^ Penicillin allergy is frequently self-reported by patients on the basis rashes and/or gastrointestinal symptoms, and although 8% of the population in the USA carries a history of penicillin allergy, <1 in 20 were really allergic when tested.^43^ Unverified penicillin allergy could represent a public health problem leading to use of unnecessary broad-spectrum antibiotics. Our results support the fact that there is still potential to further reduce macrolide prescribing in Norway.

### Strengths and limitations

The major strength of our study is the size of the dataset covering the entire Norwegian population. Linking data from different national registries by individual personal identification number gives the opportunity to obtain information across the public health system. However, the KUHR database does not receive any compensation claims from hospitals, nursing homes or commercial health care providers. There are currently no available statistics regarding the use of commercial primary care providers in Norway, but it is so far considered to be of negligible size.

To include all acute RTI episodes, we obtained all registered contacts, including phone and electronic communication. The diagnoses were obtained from the national reimbursement scheme that contain only one or two recorded diagnoses per claim, which in turn relies on the coding practices of the GPs. The ICPC-2 coding is necessary to generate compensation claims, and is not intended for research. A limitation of the KUHR database, compared to data from patient records, is the lack of information on clinical presentation and patient comorbidity. However, a reasonably good correspondence between medical record text and diagnosis coding in Norwegian general practice has been reported.^44^

NorPD data includes dispensed antibiotics only, and the number of prescriptions not dispensed therefore remains unknown. That 8% of the prescriptions were dispensed between day 3 and 7 may represent delayed prescriptions. Considering that only about 60% of delayed prescriptions are filled at pharmacies, compared to 92% of ordinary antibiotic prescriptions,^7^ the overall reduction in antibiotic prescriptions for RTIs may partly be attributed to more use of delayed prescribing.

By only including antibiotics dispensed the first week after a GP-diagnosed RTI, we probably reduced the risk of including antibiotics given for other indications. However, the NorPD does not contain diagnoses for each prescription. That we only included the first prescription per RTI episode, may have led to an overestimation of narrow-spectrum penicillin if patients were prescribed a second antibiotic in cases of treatment failure.

The NorPD only contains information on strength and size of the dispensed package of antibiotic, and no information about the prescribed dose or duration of the antibiotic course. We have, therefore, reported DDD per prescription as a standardized estimate of treatment dose and duration for each prescription. For penicillins, we observed increased mean DDDs per prescription during the study period. However, the DDD value defined by the WHO does not necessarily reflect the prescribed daily doses, as the dose vary according to diagnosis and patient characteristics.^45^ The definition of one DDD for PcV is 2 g,^23^ whereas the Norwegian primary care guidelines for treatment of pneumonia in adults recommend 4 g for 7 days, resulting in a prescription of 14 DDD.^12^ The DDD definition for macrolides and tetracyclines correspond better to the guideline recommendations. Thus, a shift towards more use of PcV would lead to increased mean DDD per prescription without necessarily reflecting enlarged prescribed doses.

### Implications for policy and practice

The decreased rate of prescriptions per episode indicates that GPs’ prescribing practices contribute to the overall reduction in antibiotic consumption reported by the national surveillance programme.^9^

So far, efforts to improve prescribing in Norwegian general practice have been time limited and project based. Our findings may reflect effects of these interventions, as reduced prescribing per RTI episode and increased use of PcV indicate an increased adherence to the national guidelines.^8,12^ The reduction in RTI antibiotic prescribing was substantially larger than the overall reduction in antibiotic use reported in Norway during the period,^9^ demonstrating that the potential for change in antibiotic use for RTIs is larger than for other infections. To ensure further improvement of antibiotic use, it is important to establish a permanent system for quality improvement of antibiotic prescribing in general practice. It is also crucial to assess whether reducing antibiotic treatment too much may have undesirable effects such as increased rates of complications.

### Conclusion

This study demonstrates stable RTI episode rates and decreased antibiotic prescription rates for RTIs for adults in Norwegian general practice during 2012–2019. We also observed a shift towards relatively more use of phenoxymethylpenicillin and less use of broad-spectrum antibiotics. These changes are in line with the aims of the Norwegian AMR strategy.

## Supplementary Material

dlac135_Supplementary_Data

## Supplementary data

Table S1 is available as Supplementary data at *JAC-AMR* online.
