# Supplementary material for: Antibiotic treatment of respiratory tract infections in adults in Norwegian general practice
Source: JAC Antimicrob Resist. 2023 Jan 7;5(1):dlac135. doi: 10.1093/jacamr/dlac135 (PMC9825809; doi:10.1093/jacamr/dlac135)
Supplement: dlac135_Supplementary_Data [file dlac135_supplementary_data.docx]

| *Diagnosis* | *Study mean* | *2012* | *2013* | *2014* | *2015* | *2016* | *2017* | *2018* | *2019* | *Mean annual change ^†^* | *( 95% CI )* | *Rank* |
| --- | --- | --- | --- | --- | --- | --- | --- | --- | --- | --- | --- | --- |
| **R04 Breathing problem other** |  |  |  |  |  |  |  |  |  |  |  | 1 |
| Episode rate ^a^ | 3 | 3 | 3 | 3 | 3 | 3 | 3 | 3 | 4 | 0.00 | (-0.05 to 0.05) |  |
| Antibiotic prescription rate ^b^ | 2 | 3 | 3 | 3 | 2 | 2 | 2 | 2 | 2 | -0.24 | (-0.30 to -0.17)* |  |
| PcV proportion (%) ^c^ | 24 | 26 | 27 | 18 | 24 | 24 | 27 | 23 | 28 | 0.24 | (-1.05 to 1.52) |  |
| **R07 Sneezing/nasal congestion** |  |  |  |  |  |  |  |  |  |  |  | 2 |
| Episode rate | 4 | 3 | 3 | 4 | 4 | 4 | 4 | 5 | 5 | 0.27 | (0.23 to 0.31)* |  |
| Antibiotic prescription rate | 3 | 3 | 3 | 3 | 3 | 3 | 2 | 2 | 2 | -0.23 | (-0.28 to -0.19)* |  |
| PcV proportion (%) | 29 | 28 | 26 | 26 | 27 | 27 | 33 | 32 | 33 | 0.93 | (0.22 to 1.64)* |  |
| **R23 Voice symptom** |  |  |  |  |  |  |  |  |  |  |  | 3 |
| Episode rate | 18 | 2 | 2 | 2 | 2 | 2 | 2 | 2 | 2 | 0.03 | (0.01 to 0.05)* |  |
| Antibiotic prescription rate | 4 | 5 | 5 | 4 | 4 | 3 | 3 | 2 | 3 | -0.46 | (-0.56 to -0.36)* |  |
| PcV proportion (%) | 24 | 20 | 24 | 22 | 23 | 22 | 26 | 23 | 32 | 1.02 | (-0.11 to 2.14) |  |
| **R25 Sputum/Phlegm abnormal** |  |  |  |  |  |  |  |  |  |  |  | 4 |
| Episode rate | 0 | 0 | 0 | 0 | 0 | 0 | 0 | 0 | 0 | 0.00 | (0.00 to 0.00) |  |
| Antibiotic prescription rate | 5 | 4 | 5 | 6 | 7 | 3 | 4 | 5 | 4 | -0.14 | (-0.69 to 0.40) |  |
| PcV proportion (%) | 18 | 27 | 31 | 5 | 17 | 22 | 21 | 13 | 20 | -1.05 | (-4.19 to 2.10) |  |
| **R08 Nose symptom** |  |  |  |  |  |  |  |  |  |  |  | 5 |
| Episode rate | 2 | 2 | 2 | 2 | 2 | 2 | 2 | 2 | 2 | 0.02 | (-0.14 to 0.06) |  |
| Antibiotic prescription rate | 6 | 7 | 7 | 7 | 7 | 6 | 5 | 5 | 5 | -0.37 | (-0.56 to -0.18)* |  |
| PcV proportion (%) | 24 | 27 | 22 | 23 | 24 | 25 | 22 | 23 | 28 | 0.06 | (-0.86 to 0.98) |  |
| **R03 Shortness of breath** |  |  |  |  |  |  |  |  |  |  |  | 6 |
| Episode rate | 0 | 0 | 0 | 0 | 0 | 0 | 0 | 0 | 0 | 0.00 | (-0.00 to 0.00) |  |
| Antibiotic prescription rate | 5 | 7 | 6 | 6 | 7 | 2 | 2 | 3 | 3 | -0.76 | (-1.27 to -0.25)* |  |
| PcV proportion (%) | 22 | 22 | 19 | 23 | 25 | 29 | 14 | 17 | 25 | -0.12 | (-2.07 to 1.82) |  |
| **R02 Wheezing** |  |  |  |  |  |  |  |  |  |  |  | 7 |
| Episode rate | 16 | 15 | 15 | 15 | 16 | 17 | 17 | 17 | 18 | 0.40 | (0.29 to 0.52)* |  |
| Antibiotic prescription rate | 6 | 7 | 7 | 6 | 6 | 5 | 5 | 5 | 5 | -0.39 | (-0.48 to -0.30)* |  |
| PcV proportion (%) | 22 | 20 | 19 | 21 | 22 | 22 | 24 | 23 | 24 | 0.71 | (0.50 to 0.91)* |  |
| **R01 Pain respiratory system** |  |  |  |  |  |  |  |  |  |  |  | 8 |
| Episode rate | 0 | 1 | 0 | 0 | 0 | 0 | 0 | 0 | 0 | -0.32 | (-0.04 to -0.03)* |  |
| Antibiotic prescription rate | 6 | 7 | 8 | 7 | 6 | 6 | 5 | 6 | 6 | -0.31 | (-0.52 to -0.11)* |  |
| PcV proportion (%) | 36 | 32 | 34 | 34 | 41 | 41 | 31 | 37 | 45 | 1.15 | (-0.62 to 2.92) |  |
| **R80 Influenza** |  |  |  |  |  |  |  |  |  |  |  | 9 |
| Episode rate | 19 | 19 | 22 | 13 | 20 | 20 | 18 | 26 | 16 | 0.18 | (-1.33 to 1.70) |  |
| Antibiotic prescription rate | 7 | 9 | 8 | 8 | 7 | 7 | 6 | 5 | 5 | -0.58 | (-0.70 to -0.46)* |  |
| PcV proportion (%) | 41 | 35 | 39 | 39 | 39 | 43 | 43 | 45 | 44 | 1.23 | (0.74 to 1.72)* |  |
| **R05 Cough** |  |  |  |  |  |  |  |  |  |  |  | 10 |
| Episode rate | 37 | 41 | 37 | 35 | 37 | 37 | 37 | 35 | 34 | -0.61 | (-1.24 to 0.02) |  |
| Antibiotic prescription rate | 13 | 20 | 16 | 15 | 14 | 12 | 10 | 9 | 9 | -1.48 | (-1.81 to -1.15)* |  |
| PcV proportion (%) | 20 | 16 | 19 | 19 | 20 | 21 | 23 | 24 | 24 | 1.09 | (0.85 to 1.32)* |  |
| **R29 Respiratory symptom other** |  |  |  |  |  |  |  |  |  |  |  | 11 |
| Episode rate | 3 | 4 | 3 | 3 | 3 | 3 | 2 | 2 | 2 | -0.21 | (-0.25 to -0.16)* |  |
| Antibiotic prescription rate | 15 | 21 | 18 | 15 | 15 | 13 | 11 | 11 | 12 | -1.39 | (-1.90 to -0.89)* |  |
| PcV proportion (%) | 24 | 21 | 24 | 26 | 26 | 24 | 25 | 25 | 24 | 0.27 | (-0.33 to 0.87) |  |
| **R21 Throat symptom** |  |  |  |  |  |  |  |  |  |  |  | 12 |
| Episode rate | 15 | 16 | 15 | 15 | 15 | 15 | 15 | 14 | 15 | -0.09 | (-0.24 to 0.06) |  |
| Antibiotic prescription rate | 15 | 20 | 19 | 18 | 17 | 15 | 13 | 12 | 11 | -1.34 | (-1.54 to -1.15)* |  |
| PcV proportion (%) | 66 | 64 | 64 | 66 | 66 | 67 | 69 | 69 | 70 | 0.84 | (0.70 to 0.98)* |  |
| **H01 Ear pain** |  |  |  |  |  |  |  |  |  |  |  | 13 |
| Episode rate | 2 | 2 | 2 | 3 | 3 | 3 | 2 | 2 | 2 | -0.02 | (-0.09 to 0.05) |  |
| Antibiotic prescription rate | 15 | 19 | 19 | 17 | 16 | 15 | 13 | 13 | 13 | -0.99 | (-1.29 to -.70)* |  |
| PcV proportion (%) | 56 | 54 | 58 | 56 | 54 | 54 | 60 | 60 | 57 | 0.48 | (-0.35 to 1.31) |  |
| **R74 URTI** |  |  |  |  |  |  |  |  |  |  |  | 14 |
| Episode rate | 65 | 63 | 59 | 58 | 63 | 65 | 70 | 71 | 75 | 2.20 | (1.06 to 3.33)* |  |
| Antibiotic prescription rate | 18 | 24 | 22 | 21 | 19 | 17 | 15 | 14 | 14 | -1.61 | (-1.94 to -1.27)* |  |
| PcV proportion (%) | 56 | 50 | 54 | 54 | 55 | 57 | 60 | 62 | 62 | 1.68 | (1.32 to 2.05)* |  |
| **R77 Laryngitis/tracheitis** |  |  |  |  |  |  |  |  |  |  |  | 15 |
| Episode rate | 1 | 1 | 1 | 1 | 1 | 1 | 1 | 1 | 1 | -0.04 | (-0.05 to -0.03)* |  |
| Antibiotic prescription rate | 21 | 28 | 26 | 23 | 21 | 18 | 16 | 15 | 16 | -1.94 | (-2.48 to -1.41)* |  |
| PcV proportion (%) | 34 | 29 | 35 | 35 | 32 | 37 | 35 | 31 | 41 | 0.82 | (-0.46 to 2.10) |  |
| **R09 Sinus symptom** |  |  |  |  |  |  |  |  |  |  |  | 16 |
| Episode rate | 5 | 5 | 5 | 5 | 5 | 6 | 6 | 5 | 6 | 0.16 | (0.08 to 0.25)* |  |
| Antibiotic prescription rate | 24 | 32 | 31 | 28 | 27 | 24 | 20 | 19 | 19 | -2.13 | (-2.50 to -1.75)* |  |
| PcV proportion (%) | 44 | 41 | 42 | 42 | 43 | 43 | 46 | 47 | 48 | 1.03 | (0.67 to 1.38)* |  |
| **R83 Respiratory infection other** |  |  |  |  |  |  |  |  |  |  |  | 17 |
| Episode rate | 16 | 23 | 19 | 15 | 14 | 13 | 13 | 15 | 18 | -0.77 | (-1.95 to 0.41) |  |
| Antibiotic prescription rate | 29 | 37 | 33 | 33 | 29 | 26 | 23 | 23 | 24 | -2.15 | (-2.82 to -1.49)* |  |
| PcV proportion (%) | 30 | 23 | 28 | 28 | 29 | 31 | 35 | 36 | 35 | -1.65 | (1.12 to 2.17)* |  |
| **R71 Whooping cough** |  |  |  |  |  |  |  |  |  |  |  | 18 |
| Episode rate | 0 | 1 | 0 | 0 | 0 | 0 | 0 | 0 | 0 | -0.03 | (-0.07 to -0.00)* |  |
| Antibiotic prescription rate | 52 | 51 | 51 | 53 | 52 | 50 | 53 | 49 | 54 | 0.15 | (-0.44 to 0.74) |  |
| PcV proportion (%) | 3 | 4 | 3 | 3 | 3 | 4 | 3 | 4 | 2 | -0.10 | (-0.29 to 0.86) |  |
| **R78 Acute bronchitis** |  |  |  |  |  |  |  |  |  |  |  | 19 |
| Episode rate | 27 | 31 | 26 | 26 | 30 | 30 | 28 | 24 | 22 | -0.82 | (-1.82 to 0.18) |  |
| Antibiotic prescription rate | 44 | 57 | 53 | 50 | 45 | 41 | 36 | 34 | 36 | -3.37 | (-4.15 to -2.59)* |  |
| PcV proportion (%) | 25 | 20 | 23 | 24 | 25 | 27 | 30 | 30 | 29 | 1.51 | (1.04 to 1.99)* |  |
| **R75 Sinusitis** |  |  |  |  |  |  |  |  |  |  |  | 20 |
| Episode rate | 26 | 32 | 30 | 27 | 27 | 25 | 24 | 22 | 22 | -1.47 | (-1.73 to -1.21)* |  |
| Antibiotic prescription rate | 57 | 65 | 64 | 61 | 59 | 55 | 51 | 49 | 49 | -2.69 | (-3.06 to -2.32)* |  |
| PcV proportion (%) | 50 | 46 | 47 | 48 | 49 | 51 | 55 | 56 | 57 | 1.74 | (1.33 to 2.15)* |  |
| **H71 Otitis media** |  |  |  |  |  |  |  |  |  |  |  | 21 |
| Episode rate | 5 | 5 | 5 | 5 | 5 | 5 | 4 | 4 | 5 | -0.11 | (-0.17 to -0.05)* |  |
| Antibiotic prescription rate | 65 | 68 | 68 | 66 | 65 | 63 | 61 | 61 | 62 | -1.17 | (-1.49 to -0.86)* |  |
| PcV proportion (%) | 70 | 66 | 66 | 68 | 68 | 71 | 73 | 74 | 75 | 1.32 | (1.05 to 1.58)* |  |
| **R81 Pneumonia** |  |  |  |  |  |  |  |  |  |  |  | 22 |
| Episode rate | 21 | 29 | 23 | 21 | 22 | 21 | 21 | 19 | 17 | -1.20 | (-1.92 to -0.47)* |  |
| Antibiotic prescription rate | 66 | 68 | 66 | 67 | 67 | 67 | 66 | 64 | 65 | -0.39 | (-0.64 to -0.13)* |  |
| PcV proportion (%) | 40 | 34 | 38 | 38 | 40 | 42 | 44 | 45 | 45 | 1.55 | (1.13 to 1.97)* |  |
| **R72+R76 Tonsillitis acute** |  |  |  |  |  |  |  |  |  |  |  | 23 |
| Episode rate | 12 | 15 | 13 | 13 | 12 | 12 | 12 | 11 | 11 | -0.48 | (-0.69 to -0.27)* |  |
| Antibiotic prescription rate | 76 | 76 | 76 | 76 | 77 | 76 | 75 | 74 | 76 | -0.21 | (-0.43 to 0.02) |  |
| PcV proportion (%) | 83 | 80 | 81 | 81 | 82 | 84 | 86 | 86 | 87 | 1.07 | (0.86 to 1.29)* |  |
